# Personality and coping as gendered predictors of distress and well-being in nursing students

**DOI:** 10.1192/j.eurpsy.2021.1074

**Published:** 2021-08-13

**Authors:** C. Laranjeira, A. Querido

**Affiliations:** Citechcare, Polytechnic of Leiria, Leiria, Portugal

**Keywords:** Distress, coping, personality, predictors

## Abstract

**Introduction:**

Previous studies about relationship between personality factors and stress related processes mainly focus on relation between these factors and application of coping strategies.

**Objectives:**

This study expanded previous research by examining the combined contribution of personality traits (NEO-FFI) and coping strategies (Brief COPE) in the prediction of stress, depressive symptoms, anxiety symptoms (DASS-21), and psychological well-being (WHO-5) among undergraduate nursing students.

**Methods:**

This cross-sectional study was performed in 2017. Participants of this study were 75 nursing students (men=37, women=38) from one Portuguese School of Health Sciences. The students who agreed to participate filled out an informed consent. Then the questionnaires were administered in a random order to avoid order effects in the data.

**Results:**

Regarding personality, women reported higher conscientiousness and agreeableness than men. There were no gender differences in coping. Among men, openness and agreeableness (inversely) and neuroticism predicted stress. In women, neuroticism and venting predicted stress. Regarding depression, conscientiousness and extraversion (inversely) and neuroticism were predictors for men, whereas neuroticism, self-blame, and denial were predictors for women. Conscientiousness and extraversion (inversely) and venting and denial predicted anxiety in men, as did neuroticism and venting in women. For well-being, conscientiousness and extraversion were predictors among men; neuroticism and seeking instrumental support (inversely) and extraversion were predictors among women. Personality traits dominated the prediction of distress and well-being in men, while both personality and coping were predictors in women.

**Conclusions:**

These findings indicate that it is not the degree of each personality trait or coping strategy but the pattern of relationship between these phenomena and psychological outcomes that is of relevance. The results could inform gendered preventive and treatment interventions for college students.

